# Identification of BGN positive fibroblasts as a driving factor for colorectal cancer and development of its related prognostic model combined with machine learning

**DOI:** 10.1186/s12885-024-12251-4

**Published:** 2024-04-23

**Authors:** Shangshang Hu, Qianni Xiao, Rui Gao, Jian Qin, Junjie Nie, Yuhan Chen, Jinwei Lou, Muzi Ding, Yuqin Pan, Shukui Wang

**Affiliations:** 1 School of Medicine, Southeast University, 210009 Nanjing, Jiangsu China; 2 General Clinical Research Center, Nanjing First Hospital, Nanjing Medical University, No. 68, Changle Road, 210006 Nanjing, Jiangsu China; 3 School of Basic Medicine and Clinical Pharmacy, China Pharmaceutical University, 211122 Nanjing, Jiangsu China; 4 Jiangsu Collaborative Innovation Center on Cancer Personalized Medicine, Nanjing Medical University, 211100 Nanjing, Jiangsu China

**Keywords:** Colorectal cancer (CRC), Cancer associated fibroblasts (CAFs), Machine learning, Prognosis

## Abstract

**Background:**

Numerous studies have indicated that cancer-associated fibroblasts (CAFs) play a crucial role in the progression of colorectal cancer (CRC). However, there are still many unknowns regarding the exact role of CAF subtypes in CRC.

**Methods:**

The data for this study were obtained from bulk, single-cell, and spatial transcriptomic sequencing data. Bioinformatics analysis, in vitro experiments, and machine learning methods were employed to investigate the functional characteristics of CAF subtypes and construct prognostic models.

**Results:**

Our study demonstrates that Biglycan (BGN) positive cancer-associated fibroblasts (BGN + Fib) serve as a driver in colorectal cancer (CRC). The proportion of BGN + Fib increases gradually with the progression of CRC, and high infiltration of BGN + Fib is associated with poor prognosis in terms of overall survival (OS) and recurrence-free survival (RFS) in CRC. Downregulation of BGN expression in cancer-associated fibroblasts (CAFs) significantly reduces migration and proliferation of CRC cells. Among 101 combinations of 10 machine learning algorithms, the StepCox[both] + plsRcox combination was utilized to develop a BGN + Fib derived risk signature (BGNFRS). BGNFRS was identified as an independent adverse prognostic factor for CRC OS and RFS, outperforming 92 previously published risk signatures. A Nomogram model constructed based on BGNFRS and clinical-pathological features proved to be a valuable tool for predicting CRC prognosis.

**Conclusion:**

In summary, our study identified BGN + Fib as drivers of CRC, and the derived BGNFRS was effective in predicting the OS and RFS of CRC patients.

**Supplementary Information:**

The online version contains supplementary material available at 10.1186/s12885-024-12251-4.

## Graphical Abstract

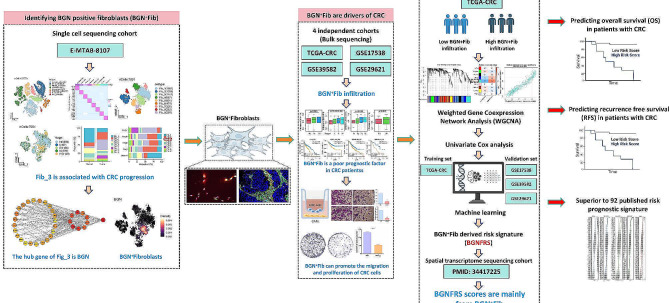

## Introduction

Colorectal cancer (CRC) is a prevalent malignancy on a global scale, with escalating incidence and mortality trends over recent decades [1]. Projections indicate that by 2030, the worldwide burden of CRC will exceed 2.2 million new cases, resulting in over 1.1 million fatalities, thus posing a substantial threat to public health [2]. Despite significant advancements in treatment methods and techniques, many patients still face the risk of recurrence and metastasis, severely affecting their prognosis and survival rates [3]. Consequently, the identification of promising therapeutic targets for CRC and the development of robust prognostic models have emerged as critical avenues of research.

The tumor microenvironment is a complex structure composed of stromal cells and extracellular matrix (ECM) components [4]. Stromal cells predominantly consist of cancer-associated fibroblasts (CAFs), endothelial cells, and various immune cells [5]. In recent years, CAFs have attracted widespread attention in the development and progression of CRC. CAFs are a key cell type in the tumor microenvironment, exerting crucial roles in the growth, metastasis, and treatment resistance of CRC through mechanisms such as cytokine secretion, regulation of the extracellular matrix, and promotion of tumor cell invasion [6]. Current research suggests that CAFs are a collection of multiple cellular subtypes, exhibiting diverse biological functions and significant heterogeneity [7]. However, there are still many unknowns regarding the exact role of CAF subtypes in CRC.

Prognostic evaluation involves predicting and categorizing patient survival period, RFS, and treatment response based on diverse clinical and pathological features [8]. Currently, prognostic evaluation in clinical practice relies primarily on clinical and pathological features such as tumor stage, histological type, and grade [9]. However, these conventional prognostic factors often lack precision in forecasting patient outcomes, prompting the need for the introduction of more predictive indicators and models [10]. Machine learning, as an emerging technique for data analysis and pattern recognition, has shown great potential in various domains [11, 12]. Particularly in the medical field, machine learning can discover hidden patterns and associations within large-scale clinical and genetic data, and construct predictive models for patient prognosis [13]. Thus, the advancement of prognostic models integrating machine learning techniques holds promise for enhancing the accuracy and reliability of prognostic evaluation in CRC.

This study identified Biglycan positive fibroblast (BGN + Fib) as a driving factor in CRC using bulk, single-cell, spatial transcriptomics, and in vitro experiments. Subsequently, a machine learning approach was employed to develop a BGN + Fib derived risk signature (BGNFRS), with the objective of improving the prognostic accuracy for OS and RFS in CRC patients.

## Methods and material

### Data source and processing

The single-cell transcriptome sequencing data for this study were obtained from the ArrayExpress database (https://www.ebi.ac.uk/biostudies/arrayexpress) (accession number: E-MTAB-8107), including 7 adjacent normal tissues and 14 CRC tissues [14]. The single-cell sequencing data were integrated, batch-corrected, quality-filtered, and visualized using the “Seurat,” “dplyr,” “stringr,” and “harmony” R packages [15, 16]. The quality control filtering criteria included the exclusion of cells with low feature counts (< 200), high feature counts (> 5000), and high mitochondrial content (> 20%). The single-cell sequencing data normalization was performed using the “LogNormalize” function in the Seurat package. The “FindVariableFeatures” function in the Seurat package was used to identify the top 2000 highly variable genes. The “RunPCA” function in the Seurat package was utilized for dimensionality reduction. The “RunTSNE” function in the Seurat package was employed for clustering analysis. The “FindAllMarkers” function in the Seurat package was applied to identify marker genes for each cell subpopulation (thresholds: min.pct = 0.25, logfc.threshold > 0.25). Cell type annotation was performed using the “SingleR” R package and the CellMarker 2.0 database [17, 18]. Single-cell pseudo-time analysis and cell communication analysis were conducted using the “Monocle 2” and “CommPath” R packages, respectively [19, 20].

The bulk sequencing data for this study were obtained from The Cancer Genome Atlas (TCGA) (https://www.cancer.gov/ccg/research/genome-sequencing/tcga) and Gene Expression Omnibus (GEO) (https://www.ncbi.nlm.nih.gov/geo/) databases. The TCGA-CRC cohort included TCGA-COAD and TCGA-RED datasets. In the GEO cohort, datasets with OS and RFS information were included, including GSE17538 [21], GSE39582 [22], and GSE29621 [23].

The spatial transcriptome (ST) data were obtained from published literature [24] including two CRC samples. The ST data were processed using the “Seurat” R package. The “RunPCA” function in the Seurat package was used for dimensionality reduction of the spatial transcriptomics (ST) data, followed by clustering of similar ST points using the “FindNeighbors” and “FindClusters” functions in the Seurat package.

Furthermore, leveraging the BGN + Fib surface markers identified in the single-cell data of this study, we applied the “ssGSEA” algorithm from the “GSVA” R package to score bulk sequencing data and spatial transcriptome sequencing data [25].. The deconvolution method is currently widely used in multiple published literature [26–28].

Detailed information about these datasets is provided in Supplementary Table 1.

### Gene Set Variation Analysis(GSVA)

The scoring of gene sets was estimated using the “ssGSEA” algorithm from the “GSVA” R package [25]. The “HALLMARK” and “KEGG” gene sets used in this study were obtained from the Molecular Signatures Database (https://www.gsea-msigdb.org/gsea/msigdb).

### Gene Ontology (GO)/Kyoto Encyclopedia of genes and genomes (KEGG) and Gene Set Enrichment Analysis (GSEA)

For the analysis of GO/KEGG in single-cell sequencing data and bulk sequencing data, the “SCP” and “clusterProfiler/org.Hs.eg.db” R packages were used, respectively [29]. Gene Set Enrichment Analysis (GSEA) was performed using the “clusterProfiler/org.Hs.eg.db” and “enrichplot” R packages [29]. Enrichment terms with adjusted *P* < 0.05 were considered statistically significant.

### Prognostic analysis

Kaplan-Meier curves and univariate/multivariate Cox analysis in this study were conducted using the “survival” and “survminer” R packages. Kaplan-Meier curves were constructed by stratifying the data into high and low expression groups according to the median expression values. The “ggplot2” R package was used for visualization.

### Tumor Immune single-cell hub 2 (TISCH2)

Based on the CRC dataset in the TISCH2 database (http://tisch.comp-genomics.org/home/), the expression levels of BGN were evaluated in various cell types of CRC, including CRC_EMTAB8107, CRC_GSE108989, CRC_GSE146771, CRC_GSE166555, and CRC_GSE179784 [30]..

### Protein Interaction Network (PPI)

The protein-protein interaction (PPI) network was constructed based on the STRING database (https://cn.string-db.org/) and visualized using Cytoscape [31]. Identification of hub genes was performed utilizing the Degree algorithm.

### Weighted Gene Coexpression Network Analysis (WGCNA)

Based on the TCGA-CRC cohort, CRC samples were stratified into high BGN + Fib infiltration and low BGN + Fib infiltration groups based on the median values of BGN + Fib infiltration. The “WGCNA” R package was used to identify co-expression modules in the high BGN + Fib infiltration group [32]. Initially, we utilized soft-threshold (Set soft threshold to 5) and gene-gene correlation matrices to construct an adjacency matrix, depicting the degree of interconnection between nodes. Subsequently, we transformed the adjacency matrix into a Topological Overlap Matrix (TOM). Following this step, hierarchical clustering of genes was performed, and a dendrogram was generated to identify co-expression modules. Finally, we computed Module Eigengenes (ME) and assessed the correlation between ME and BGN + Fib, thereby identifying modules associated with BGN + Fib.

### Machine learning

This study employed ten machine learning algorithms, including Random Survival Forest (RSF), Elastic Net (Enet), Lasso, Ridge, Stepwise Cox, CoxBoost, Cox Partial Least Squares Regression (plsRcox), Supervised Principal Component (SuperPC), Generalized Boosted Regression Modeling (GBM), and Survival Support Vector Machine (Survival SVM). Based on the integration of 101 algorithm combinations from these 10 machine algorithms [28], the signature constructed in this study follows the process outlined below: (a) Based on modules identified by WGCNA, genes related to CRC prognosis were selected through single-factor Cox analysis within the module; (b) Batch correction and normalization were performed on the TCGA-CRC expression profile and the expression profiles from the GEO datasets (GSE17538, GSE39582, GSE29621). (c) Using the TCGA-CRC cohort as the training set and the remaining three cohorts (GSE17538, GSE39582, GSE29621) as validation sets, 101 algorithm combinations were applied to the prognosis-related genes; (d) For each algorithm combination, the C-index was calculated in both the validation and training sets, with the combination showing the highest average C-index considered the best. The C-index, a metric used to evaluate model performance, is commonly employed to assess the predictive accuracy of survival analysis models. The C-index ranges from 0 to 1, with values closer to 1 indicating better predictive performance [33].

### Collection and comparison of published risk signature

As of October 2023, this study retrieved published risk features from the PubMed database system using the keyword “CRC risk signature.” A total of 550 articles were retrieved, among which 150 articles constructed signatures for predicting the prognosis of CRC patients. Therefore, this study included these 150 risk signatures. By matching the expression profiles and survival information of the validation set (TCGA-CRC) and training sets (GSE17538, GSE39582, GSE29621), the C-index of these risk signatures was calculated, and the prognostic value of BGNFRS was ultimately compared with these risk signatures. Among these 150 risk signatures, only the expression profiles of 92 signature genes could be matched to our training and validation sets.

### Nomogram prediction model

The Nomogram prediction model was constructed using the “survival,” “regplot,” and “rms” R packages [34]. The parameters of this prediction model included BGNFRS risk score, Gender, T, M, N, and stage. The predictive accuracy of the Nomogram prediction model and other parameters was evaluated using the time-dependent ROC curve constructed using the “timeROC” R package.

### Tissue sample collection

In this study, paraffin-embedded tissue sections from 3 pairs of tumors and adjacent non-cancer tissues were collected and utilized for detecting the expression of BGN. The clinical characteristics of the 3 patients were as follows: Patient 1 (age: 70, male, TNM stage: T4aN1bM0), Patient 2 (age: 57, female, TNM stage: T3N0M0), and Patient 3 (age: 68, female, TNM stage: T1N0M0). All patients provided informed consent and had not received any prior anti-tumor treatment. Additionally, all patients were pathologically diagnosed with CRC. The Ethics Committee of Nanjing First Hospital, Nanjing Medical University approved the use of all human specimens.

### Isolating fibroblasts

In this study, well-separated Normal fibroblasts (NFs) and CAFs isolated by our research group were used. The methods for fibroblast isolation and identification can be found in our previous study [35]. Fibroblasts were obtained from fresh tissues following established protocols. In brief, the tissue samples were finely minced and subjected to digestion using a mixture of 1 mg/mL collagenase (cat. #C4-BIOC, Sigma-Aldrich), Dulbecco’s modified Eagle’s medium (DMEM; cat. #KGM12800, KeyGen), and 10% fetal bovine serum (FBS; cat. #12,106 C, Sigma, Sigma-Aldrich) for 2 h at 37 °C with agitation. Following centrifugation, the cell pellets were resuspended and filtered through a 100 μm cell strainer. Subsequently, the cells were cultured in DMEM supplemented with 10% FBS. After 2 h, the culture medium was replaced. Notably, fibroblasts exhibit a higher affinity for adherence to culture dishes compared to other cell types. The fibroblasts were characterized by the presence of two positive markers (α-SMA and Vimentin) and the absence of two negative markers (KRT20 and Desmin).

### Cell culture and transient transfection

CRC cells (HCT116 and DLD1) were cultured in DMEM complete medium (Procell, China) containing 10% fetal bovine serum. CAFs and NFs were extracted from cancer tissue and adjacent non-cancer tissue of CRC patients and cultured using fibroblast expansion basal medium (Thermo Fisher, China). All cell lines were maintained in a 37℃ and 5.0% CO2 incubator. The transfection reagent used in this study was riboFECTTMCP (Ribobio, China). Transfection experiments were performed according to the manufacturer’s instructions when the CAF density reached 60% during the cell transfection process. Ribobio, China, constructed si-BGN. siBGN_1: GGAGAACAGTGGCTTTGAA. siBGN_2: CCATCCAGTTTGGCAACTA.

### Total RNA extraction and quantitative real-time polymerase chain reaction (qRT-PCR)

Total RNA was extracted in this study using the Trizol Kit (Vazyme, China). Reverse transcription and quantitative fluorescent PCR were performed using HiScript III RT SuperMix for qPCR and SYBR Green PCR Master Mix Kit (Vazyme, China). The reagent manufacturer’s instructions performed the operation process. GAPDH served as the internal reference gene for this experiment. The relative expression of genes was calculated using a 2-ΔΔCq method. Human BGN forward primer: 5’- CAGTGGCTTTGAACCTGGAG-3’. Human BGN reverse primer: 5’-GGGAGGTCTTTGGGGATGC-3’.

### Tissue immunofluorescence assay

Paraffin embedded tissue sections were deparaffinized, and antigen retrieval and Donkey serum blocking were performed. Subsequent primary antibody incubation was performed at 4 ° C overnight. Primary antibodies included a-SMA (1:400; 48,938; cell signaling technology, Ma, USA) and BGN (1:200; A5770, ABclonal, China). Secondary antibody incubation was performed the following day at a room temperature environment. Secondary antibodies included Goat anti-rabbit IgG H & L (Alexa fluor ® 647) (1:500, AB150079, Abcam, Cambridge, UK) and goat anti-mouse IgG (H + L) Alexa Fluor 488 (1:500, AB150113. Abcam, Cambridge, UK). Moreover, finally mounted with an UltraCruz mounting medium (sc-24,941, Santa Cruz Biotechnology, TX, USA) containing DAPI. Immunofluorescence signals were photographed by fluorescence microscopy (Zeiss).

### Cellular immunofluorescence experiments

First, make cell climbing slices. Subsequently, cell climbing slides were fixed, permeabilized, and blocked with Donkey Serum. Subsequent primary antibody incubation was performed at 4 ° C overnight. Secondary antibody incubation was performed the following day at a room temperature environment—final mounting with UltraCruz mounting medium containing DAPI. Immunofluorescence signals were photographed by fluorescence microscopy. Primary and secondary antibodies and dilution ratios used for cell immunofluorescence experiments were the same as for tissue immunofluorescence experiments.

### Transwell

First, starvation treated cells using an incomplete medium (without serum) the night before. Then 100 UL containing 3 × 104 cells with the incomplete medium were seeded in the upper Transwell chamber (3422, costar, USA), and 600 UL of the lower chamber containing 2 × Complete medium of 104 CAFs cells. Fixed staining was performed after 48 h of culture, and six randomly selected fields were counted for the number of migrated or invaded cells.

### Cell clone formation experiment

The experiment was divided into two groups (experimental group and NC group). The experimental group used culture medium with conditioned media from cancer-associated fibroblasts (CAF) supplemented with siBGN, while the NC group used culture medium with conditioned media from CAF without siBGN. The process of cell clone formation involved digesting cells in logarithmic growth phase with trypsin, resuspending them in cell culture medium, and counting them. The cells were then seeded in a 6-well plate with 500 cells per well and cultured for two weeks. After two weeks, staining and counting were performed.

### Statistical analysis

All statistical analyses in this study were conducted using R software version 4.3.0. Student’s t-test or Wilcoxon rank-sum test is utilized to compare continuous variables between two groups, while one-way analysis of variance or Kruskal-Wallis test is employed for differential comparisons among three groups. The log-rank test was used for comparing survival differences between the two groups. Spearman’s test was used for correlation analysis. All in vitro experiments were repeated three times. In the calculation of cell numbers in the Transwell and cell clone formation experiments, we utilized the image analysis software ImageJ. In essence, experimental images were imported into the ImageJ software, where cells were identified and counted by setting a threshold. Additionally, manual inspection and correction were carried out to ensure the accuracy and reliability of the cell count. A *p*-value less than 0.05 was considered statistically significant (*p < 0.05, **p < 0.01, ***p < 0.001).

## Result

### CAFs is the cell population with the highest communication weight, and CAFs is a poor prognostic factor for CRC

**Fig. 1:**
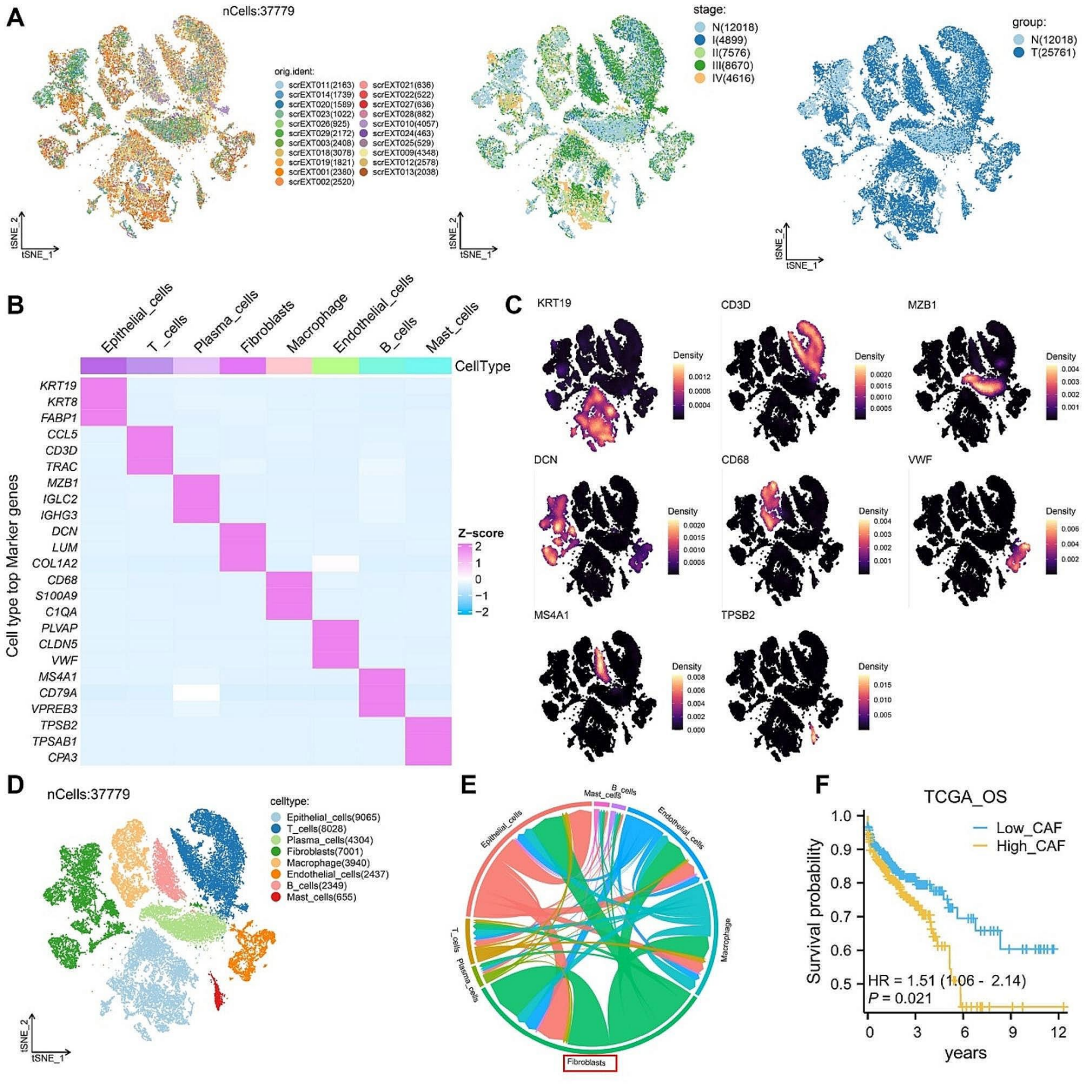
CAFs have the highest communication weight and are associated with poor prognosis in CRC **A.** T-SNE plots for each sample, different stages, and different groups. **B.** Heatmap of the top three markers for each of the 8 cell types. **C.** T-SNE plots showing the expression of specific markers for each of the 8 cell types. **D.** T-SNE plots showing the distribution of the 8 cell types. **E.** Circular plot illustrating the communication weights and strengths among the 8 cell types. **F.** Overall survival (OS) difference between high and low CAF infiltration groups

This study integrated 21 single-cell transcriptome sequencing data, including 7 normal samples and 14 tumor samples (Supplementary Table 1). Following batch correction and quality control filtering, a total of 37,779 high-quality cells were acquired. Subsequent clustering analysis of these cells showed no batch effect in the t-SNE plots for each sample, different stages, and different groups (Fig. 1A). Based on specific markers, we identified 8 cell types (Fig. 1B-D). Our results indicate a significant role of CAFs in CRC progression [36]. Therefore, CAFs were the main focus of this study. Additionally, we computed the communication weights and strengths among the 8 cell types and found that CAFs had the highest communication weight and the strongest crosstalk with epithelial cells (Fig. 1E). Due to the limited number of single-cell transcriptomic data samples in this study, we conducted deconvolution of bulk sequencing data based on surface markers of CAFs to estimate the infiltration level of CAFs in the samples. CAFs markers (LUM, DCN, COL1A1, COL1A2, FAP, PDPN, PDGFRA, PDGFRB, S100A4, ACTA2, VIM, TGFB1) were obtained from previous studies [37]. Using the TCGA-CRC cohort, CRC samples were stratified into high and low CAFs infiltration groups using the median cutoff value of CAFs infiltration. We found that high infiltration of CAFs was associated with adverse prognosis in CRC patients. (Fig. 1F).

### Identification of Biglycan (BGN) positive fibroblasts (BGN + fib) as a subgroup associated with CRC progression

**Fig. 2:**
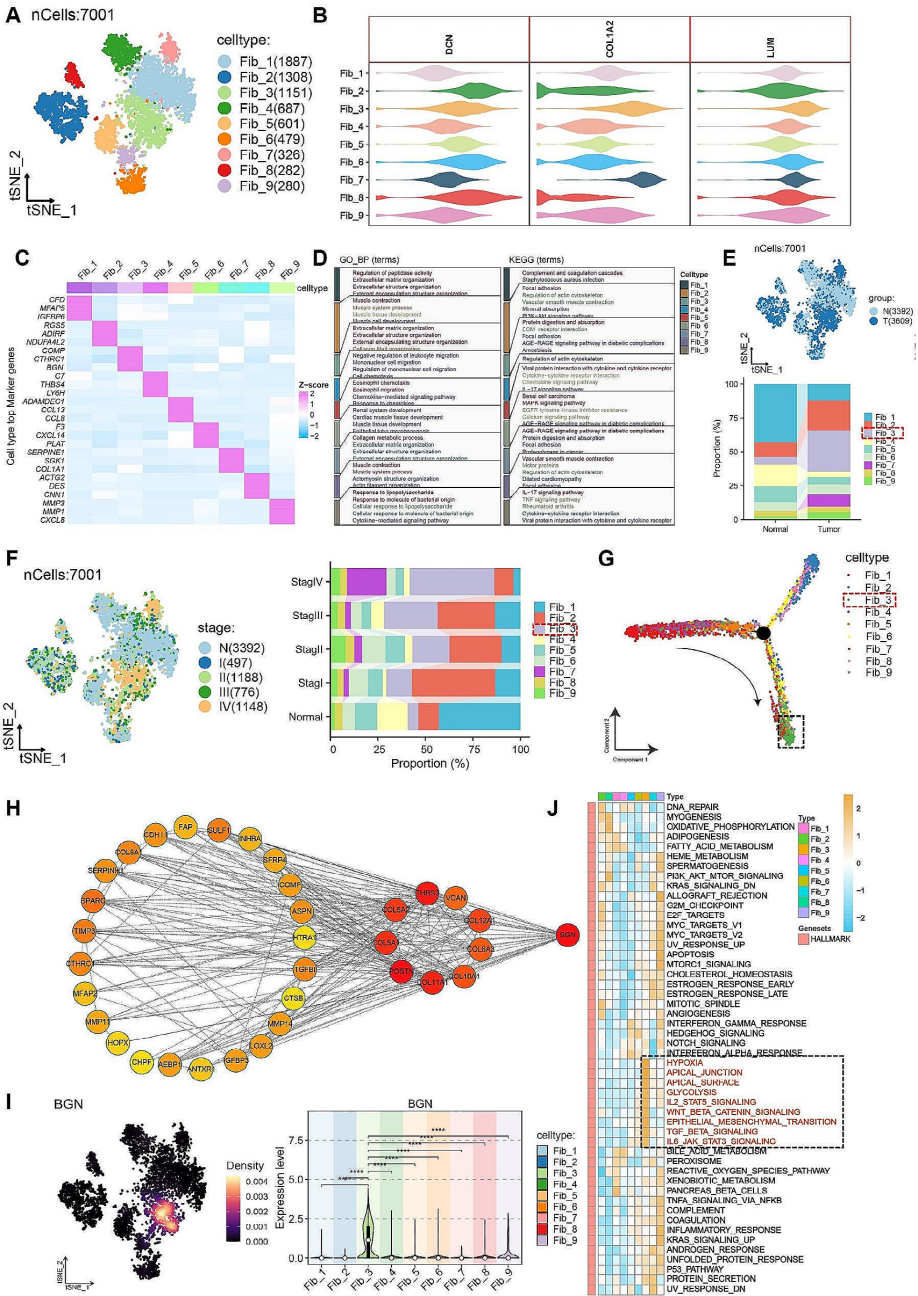
Identification of BGN + Fibroblasts (BGN + Fib) as a relevant subgroup in CRC progression **A.** Tsne plot of the nine fibroblast subgroups. **B.** Expression levels of the three fibroblast markers in the nine fibroblast subgroups. **C.** Heatmap of the top three markers for the nine fibroblast subgroups. **D.** GO/KEGG analysis of the nine fibroblast subgroups. **E.** tsne plot and proportion analysis of the normal and tumor groups. **F.** tsne plot and proportion analysis of different stages. **G.** Pseudo-time analysis. **H.** PPI network and hub gene identification of highly expressed genes (log2FC > 1, *P* < 0.05) in Fib_3. **I.** tsne plot and differential analysis of BGN expression. **J.** GSVA clustering heatmap

In this study, we conducted re-clustering and subtyping of fibroblasts based on gene expression similarity, resulting in the identification of nine fibroblast subgroups (Fib_1–9) (Fig. 2A). Each fibroblast subgroup demonstrated high expression of fibroblast markers (DCN, COLA2, and LUM) (Fig. 2B). Currently, fibroblasts are commonly classified into three types, including myofibroblasts CAF (myCAF), immune regulatory/inflammatory CAF (iCAF), and antigen presenting CAF (apCAF) [7]. Through the analysis of surface markers and biological functional characteristics of these nine fibroblast subgroups (Fig. 2C-D), Fib_1/2/3/6/8 were classified as myCAF, and Fib_4/5/7/9 were classified as iCAF. Notably, within different groups (normal and tumor), the Fib_3 subgroup exhibited the most substantial increase in the tumor group compared to the normal group and represented the highest proportion in the tumor group (Fig. 2E). Moreover, as the disease progressed, the proportion of Fib_3 increased gradually and reached the highest proportion in TNM stage IV (Fig. 2F). Subsequently pseudo-time analysis revealed that Fib_3 was in a terminal differentiation state (Fig. 2G), indicating a likely association with CRC progression. By identifying highly expressed genes (log2FC > 1, *P* < 0.05) in Fib_3, we constructed a protein-protein interaction (PPI) network to identify hub genes. Notably, BGN emerged as the most central node in this PPI network (Fig. 2H), suggesting that BGN may play an important regulatory role in Fib_3. Analysis of the expression distribution of BGN revealed predominant expression in fibroblasts (Supplementary Fig. 1A). Compared to the normal group, BGN was highly expressed in the tumor group (Supplementary Fig. 1B). Subsequent investigation into the biological attributes of BGN unveiled substantial positive associations between BGN and various signaling pathways, including epithelial-mesenchymal transition (EMT), TGF-BETA pathway, hypoxia, APICAL_JUNCTION, and IL6_JAK_STAT3 pathway, based on GSVA correlation analysis with a threshold of *R* > 6 (Supplementary Fig. 1C). Additionally, compared to the low expression group, the high expression group of BGN was associated with worse prognosis in CRC patients (Supplementary Fig. 1D). Notably, within the nine fibroblast subgroups, BGN was mainly highly expressed in Fib_3 and showed significant differences compared to other subgroups (Fig. 2I). The GSVA clustering heatmap depicted that the modules clustered by Fib_3 were highly similar to the biological characteristics of BGN (Fig. 2J). Consequently, based on these findings, Fib_3 was designated as BGN + Fibroblasts (BGN + Fib).

### BGN + fib is the driving factor of CRC progress

**Fig. 3:**
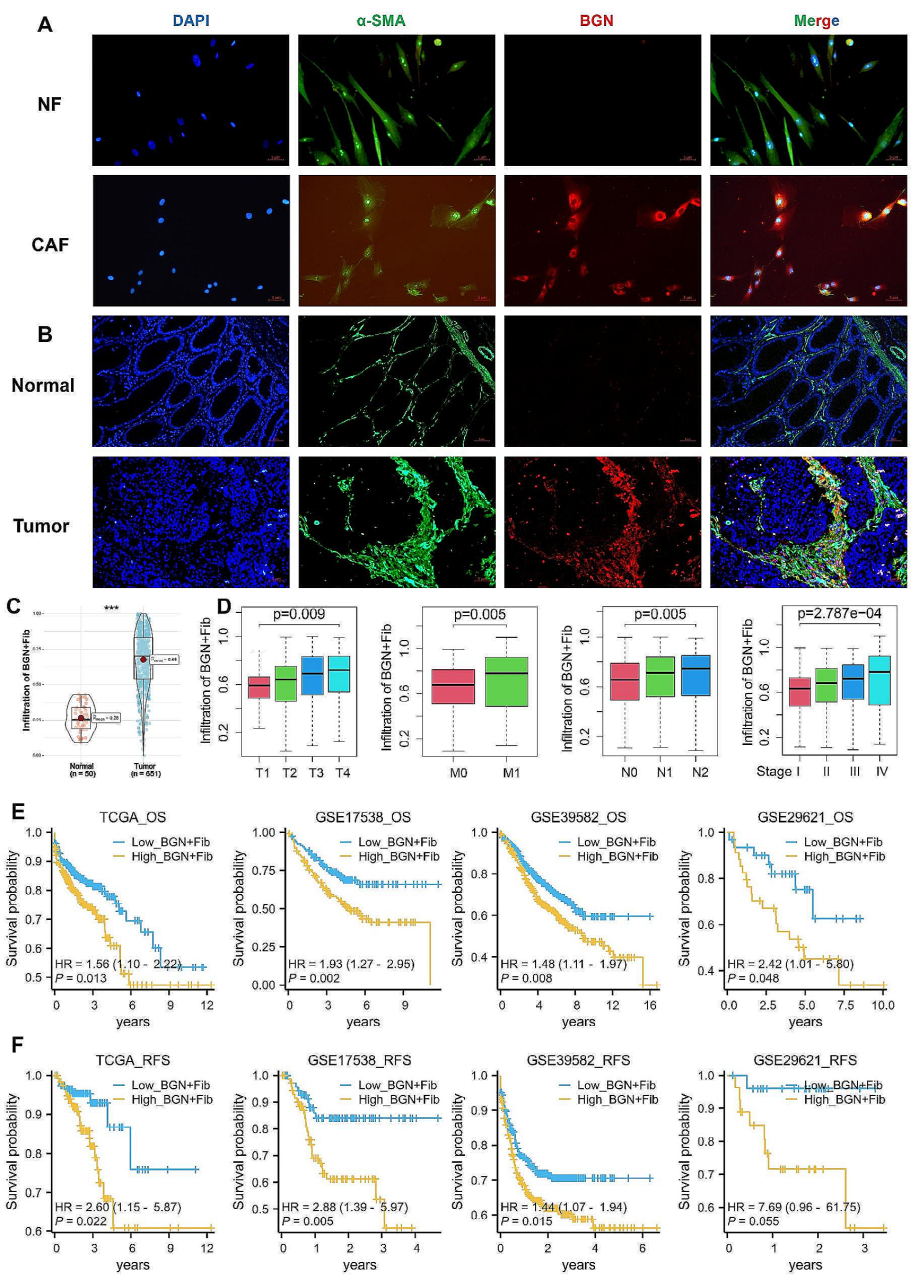
Increased expression of BGN + Fib in CRC and its association with poor prognosis and recurrence **A.** The immunofluorescence results revealed the localization of BGN and the fibroblast-specific marker (α-SMA) in both normal fibroblasts (NF) and cancer-associated fibroblasts (CAFs). **B.** The immunofluorescence results of the tissue demonstrated the localization of BGN and α-SMA in both normal colon tissue and CRC tissue. **C.** Infiltration difference of BGN + Fib in normal and tumor tissues based on the TCGA-CRC cohort. **D.** Analysis of differences in BGN + Fib infiltration and CRC pathological features (T, N, M, and stage). **E-F.** Differences in OS/RFS between high and low BGN + Fib infiltration groups

**Fig. 4:**
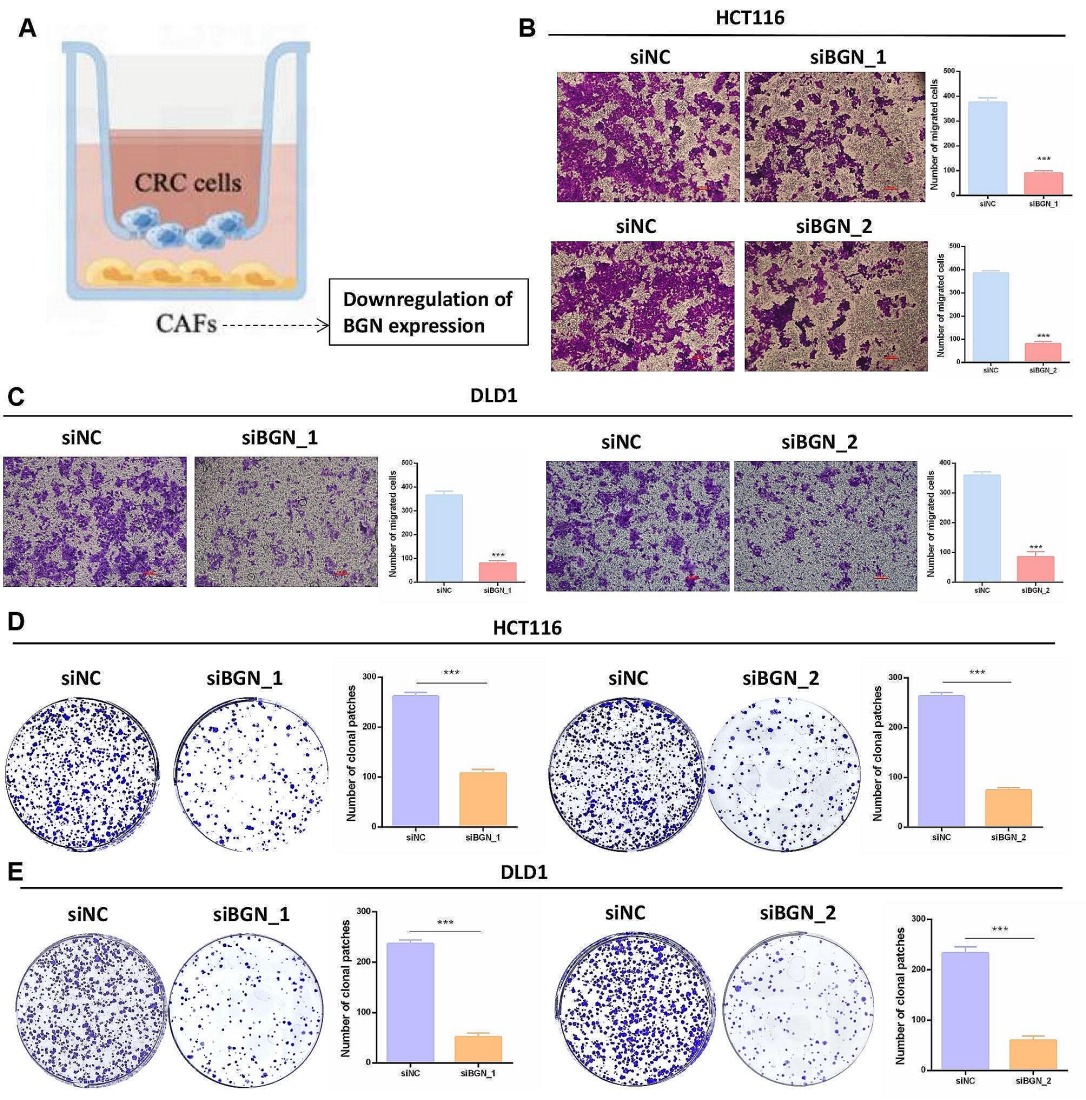
Downregulation of BGN in CAFs reduces the metastasis and proliferation of CRC cells **A.** Co-culture system of CAFs and CRC cells. **B-C.** Transwell experiment. **D-E.** Cell plate clone experiment

We isolated CAFs and NFs from fresh CRC tissues and paired normal tissues. Cell immunofluorescence results showed that BGN expression was significantly higher in CAFs compared to NFs (Fig. 3A). Tissue multiplex immunofluorescence revealed widespread presence of BGN + Fib in tumor tissues, while it was almost absent in adjacent normal tissues (Fig. 3B). Subsequently, leveraging the surface high markers of BGN + Fib (Fig. 2H) and utilizing a deconvolution algorithm, we estimated the infiltration level of BGN + Fib in bulk sequencing data from the TCGA-CRC cohort, revealing a notable increase in BGN + Fib infiltration in tumor tissues (Fig. 3C). Moreover, BGN + Fib infiltration exhibited significant differences in CRC pathological features (T, N, M, and stage), with higher grades correlating with increased BGN + Fib infiltration (Fig. 3D). Analyzing data from four cohorts with OS and RFS information (TCGA, GSE17538, GSE39582, and GSE29621), we observed a detrimental association between high BGN + Fib infiltration and poor prognosis as well as recurrence in CRC patients (Fig. 3E-F). Based on our previous analysis, we identified BGN + Fib as myCAF, which has pro-metastatic and proliferative characteristics [7]. Subsequently, we established a co-culture system of CAFs and CRC cells (Fig. 4A). By downregulating BGN expression in CAFs, and the efficiency of downregulation is shown in Supplementary Fig. 2. we observed a significant reduction in the number of metastatic CRC cells (HCT116) alongside a similar trend in another CRC cell line (DLD1) (Fig. 4B-C). In addition, downregulation of BGN expression in CAFs significantly reduced the number of colony formation in CRC cells (HCT116 and DLD1) (Fig. 4D-E). Therefore, downregulation of BGN expression also significantly decreased the proliferative effect of CRC cells. In conclusion, BGN + Fib is a driving factor in CRC progression.

### Development of BGN + fib derived risk signature (BGNFRS) combined with machine science

**Fig. 5:**
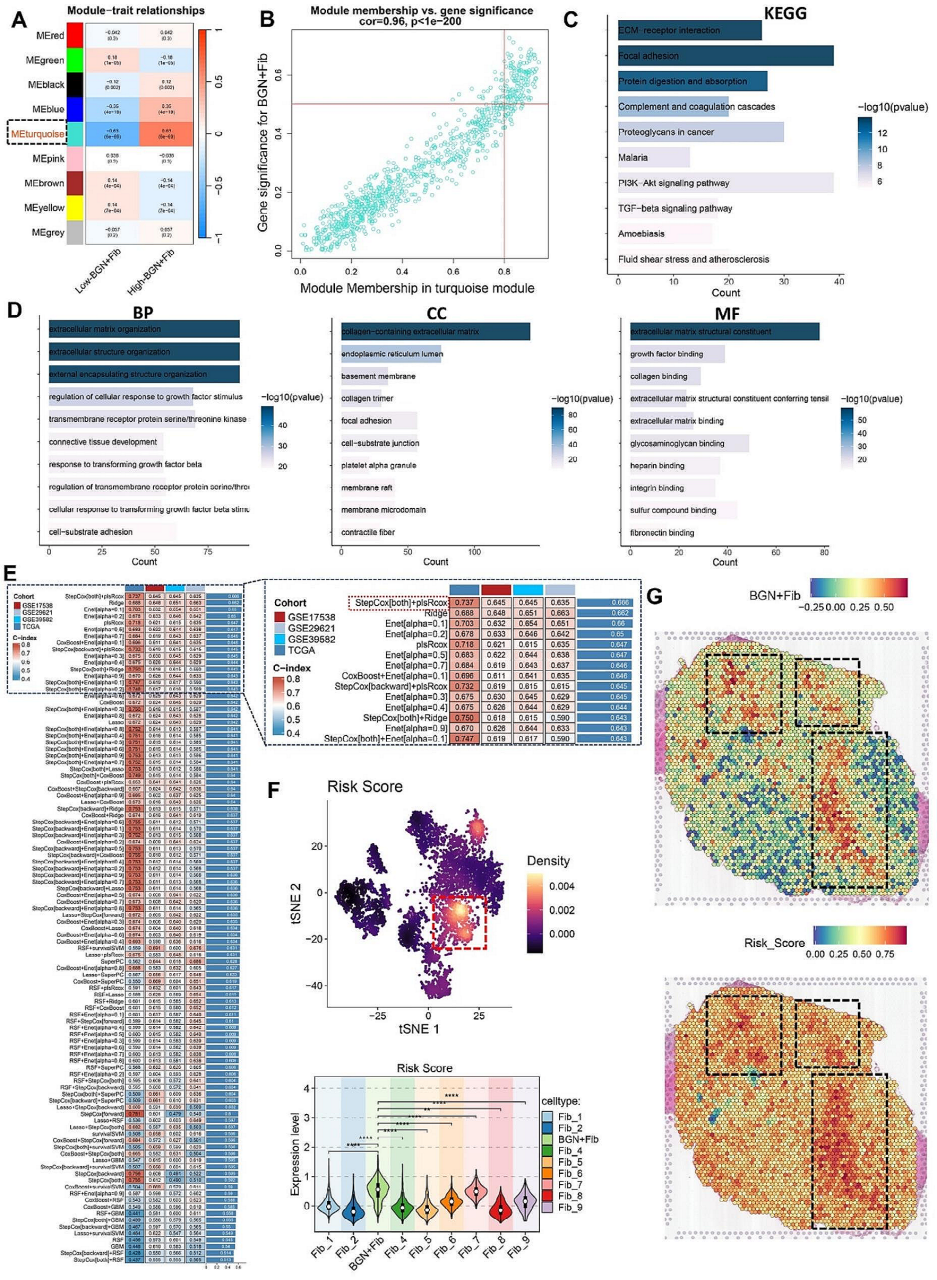
Development of BGN + Fib-related risk signature (BGNFRS) using machine learning **A.** Module correlation heatmap. **B.** Scatter plot showing the correlation between MEturquoise module and high BGN + Fib infiltration group. **C-D**. KEGG/GO analysis. **E.** Construction of 101 prediction models using various combinations of algorithms and calculation of C-index for each dataset; The C-index ranges from 0 to 1, with values closer to 1 indicating better predictive performance. **F.** Distribution and differences of BGN + Fib-related risk signature (BGNFRS) in single-cell data. **G.** Spatial localization of BGN + Fib and BGNFRS in spatial transcriptomics

In the previous analysis, we observed that high infiltration of BGN + Fib is an unfavorable factor for CRC prognosis and recurrence. Consequently, we hypothesize that genes related to BGN + Fib may have the potential to serve as a signature for assessing CRC risk. Utilizing the TCGA-CRC cohort, we segregated the samples into high BGN + Fib infiltration and low BGN + Fib infiltration group. Subsequently, we performed WGCNA and determined 9 co-expression modules when the soft threshold was set to 5 (Supplementary Fig. 3A-B). The module heatmap demonstrated that the MEturquoise module exhibited the strongest correlation with the high BGN + Fib infiltration group (Fig. 5A), displaying a correlation coefficient of 0.96 (Fig. 5B), therefore indicating superior module construction quality in the high BGN + Fib infiltration group. KEGG/GO analysis of genes in the MEturquoise module revealed their association with cell migration and extracellular matrix remodeling (Fig. 5C-D). Subsequent univariate Cox analysis of gene expression profiles within the MEturquoise module led to the identification of 167 prognostic genes (Supplementary Table 2). Leveraging the TCGA-CRC cohort as the training set, developed 101 prediction models, and assessed their performance using the C-index on validation sets (GSE17538, GSE39582, and GSE29621). Our results indicated that the combination of StepCox[both] + plsRcox had the highest average C-index (0.666) (Fig. 5E). Eventually, we obtained 17 risk gene combinations (Supplementary Table 3). Based on the expression profiles of risk genes and their corresponding risk coefficients, we calculated the risk score for each sample. In bulk sequencing data, this risk signature exhibited a strong positive correlation with BGN + Fib, demonstrating correlation coefficients exceeding 0.8 (Supplementary Fig. 3C). In single-cell sequencing data, this risk signature predominantly resided within BGN + Fib (Fig. 5F). Among the 17 signature genes, except for WNT5A and CPXM2, the other 15 signature genes mainly come from BGN + Fib (Supplementary Fig. 3D). To further confirm the spatial localization of BGN + Fib, spatial transcriptomics data were employed, revealing a substantial overlap with the distribution of the risk signature (Fig. 5G). The same pattern was observed in another spatial transcriptomics section (Supplementary Fig. 3E). Therefore, we defined this risk signature as BGN + Fib derived risk signature (BGNFRS).

### BGNFRS is an independent prognostic factor for CRC patients

**Fig. 6:**
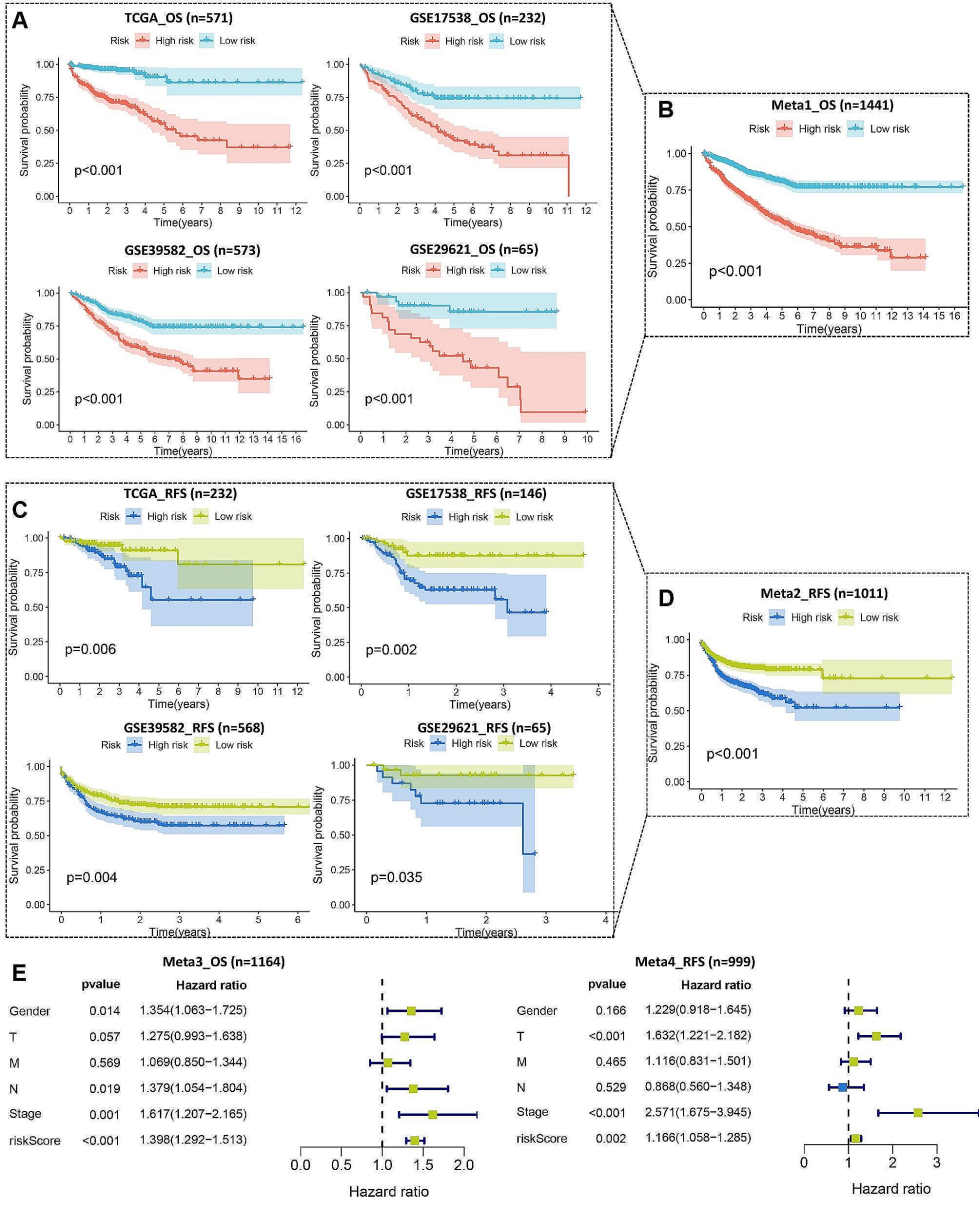
BGNFRS as an independent adverse prognostic factor for CRC patients **A.** Overall survival (OS) curves for the training set TCGA-CRC and three validation sets (GSE17538, GSE39582, and GSE29621). **B.** OS curves for the Meta1 cohort (TCGA-CRC, GSE17538, GSE39582, and GSE29621). **C.** Recurrence-free survival (RFS) curves for the four datasets. **D.** RFS curves for the Meta2 cohort (TCGA-CRC, GSE17538, GSE39582, and GSE29621). **E.** Multivariate Cox analysis combining OS/RFS with CRC clinical and pathological features for the Meta3/Meta4 cohorts (TCGA-CRC, GSE39582, and GSE29621).

CRC patients were divided into high-risk groups and low-risk groups based on the median cutoff BGNFRS. In the training set TCGA-CRC and three validation sets (GSE17538, GSE39582, and GSE29621), we observed a significant difference in OS between patients classified into high-risk and low-risk groups (*P* < 0.05) (Fig. 6A). Combining all samples from these datasets, we established the Meta1 cohort, which exhibited a consistent survival trend (*P* < 0.05) (Fig. 6B). Furthermore, our analysis of RFS across these datasets revealed a similar pattern, with patients in the high-risk group experiencing lower RFS compared to those in the low-risk group (*P* < 0.05) (Fig. 6C). Integration of all samples led to the formation of the Meta2 cohort, which also demonstrated a significant difference in RFS (*P* < 0.05) (Fig. 6D). By combining the clinical and pathological features of each dataset, we found that higher BGNFRS risk score was associated with higher clinical and pathological grade (Supplementary Fig. 4A). Multivariate Cox analysis confirmed that the BGNFRS risk score independently predicted adverse prognosis for CRC patients in terms of both OS and RFS across all datasets (Supplementary Fig. 4B-C). Notably, leveraging the comprehensive clinical and pathological features available in the TCGA-CRC, GSE39582, and GSE29621 datasets, we combined survival and clinical pathological data to establish two distinct cohorts, Meta3 and Meta4, focusing on OS and RFS, respectively. Consistently, in both the Meta3 cohort for OS and the Meta4 cohort for RFS, BGNFRS emerged as a significant independent adverse prognostic factor for CRC patients (Fig. 6E).

### Construction of nomogram prediction model to predict OS and RFS of CRC patients

**Fig. 7:**
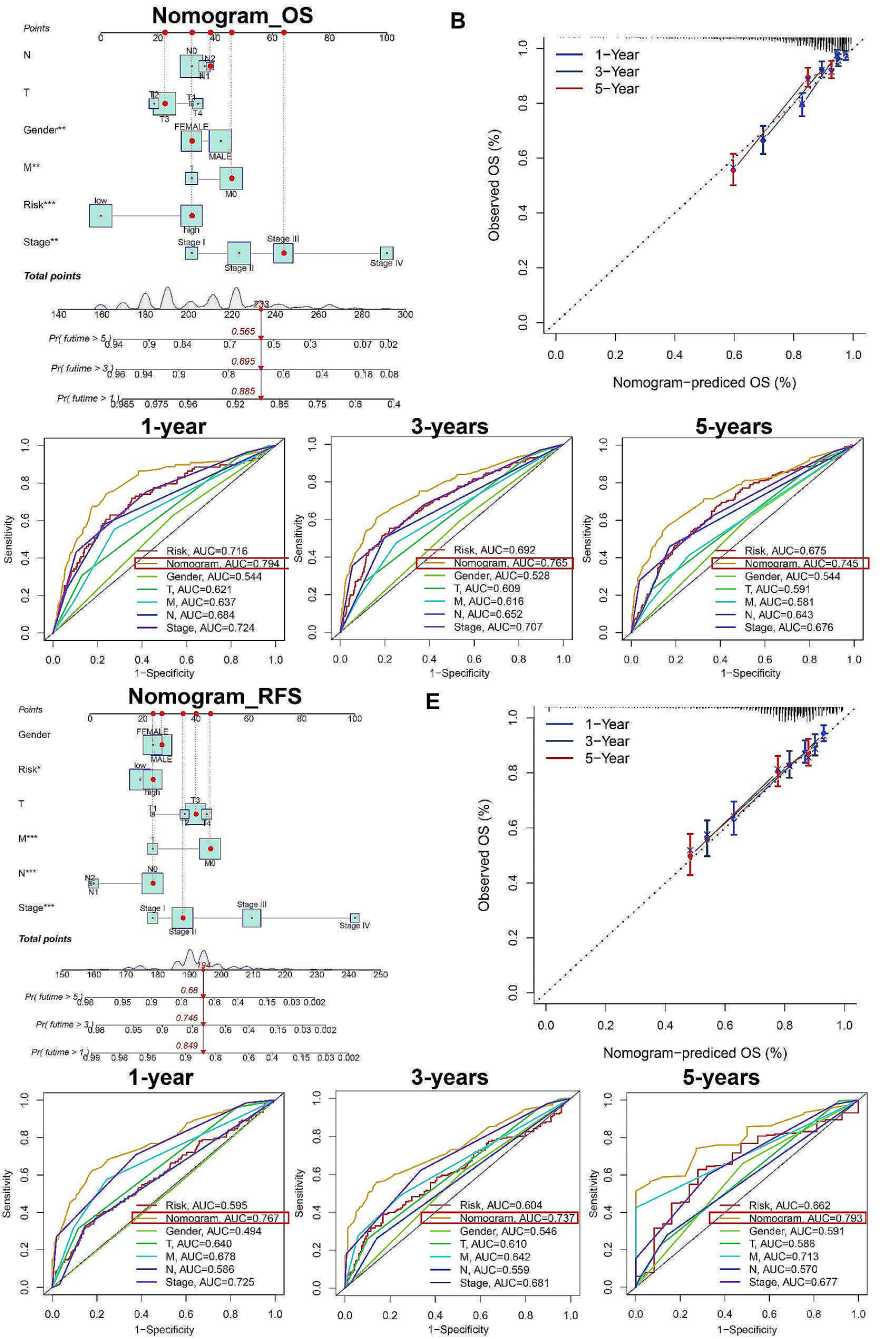
Nomogram prediction models for predicting OS and RFS in CRC patients **A.** OS nomogram prediction model constructed based on the Meta3 cohort. **B.** Calibration curve for 1, 3, and 5-year OS. **C.** ROC curve for 1, 3, and 5-year OS. **D.** RFS nomogram prediction model constructed based on the Meta4 cohort. **E.** Calibration curve for 1, 3, and 5-year RFS. **F.** ROC curve for 1, 3, and 5-year RFS.

This study categorized BGNFRS to develop a clinical prediction model for OS and RFS in CRC patients by integrating clinical and pathological features (T, N, M, stage, and gender). The OS nomogram was established using the Meta3 cohort (Fig. 7A), and the calibration curve demonstrated its robustness in predicting 1, 3, and 5-year OS (Fig. 7B). The receiver operating characteristic (ROC) curve results for 1, 3, and 5-year OS indicated that the nomogram prediction model outperformed other signature (Fig. 7C). Similarly, the RFS nomogram prediction model constructed based on the Meta4 cohort exhibited excellent predictive ability (Fig. 7D-F). Consequently, the nomogram prediction models based on BGNFRS hold significant promise for clinical utility.

### BGNFRS outperforms 92 published risk signatures

**Fig. 8:**
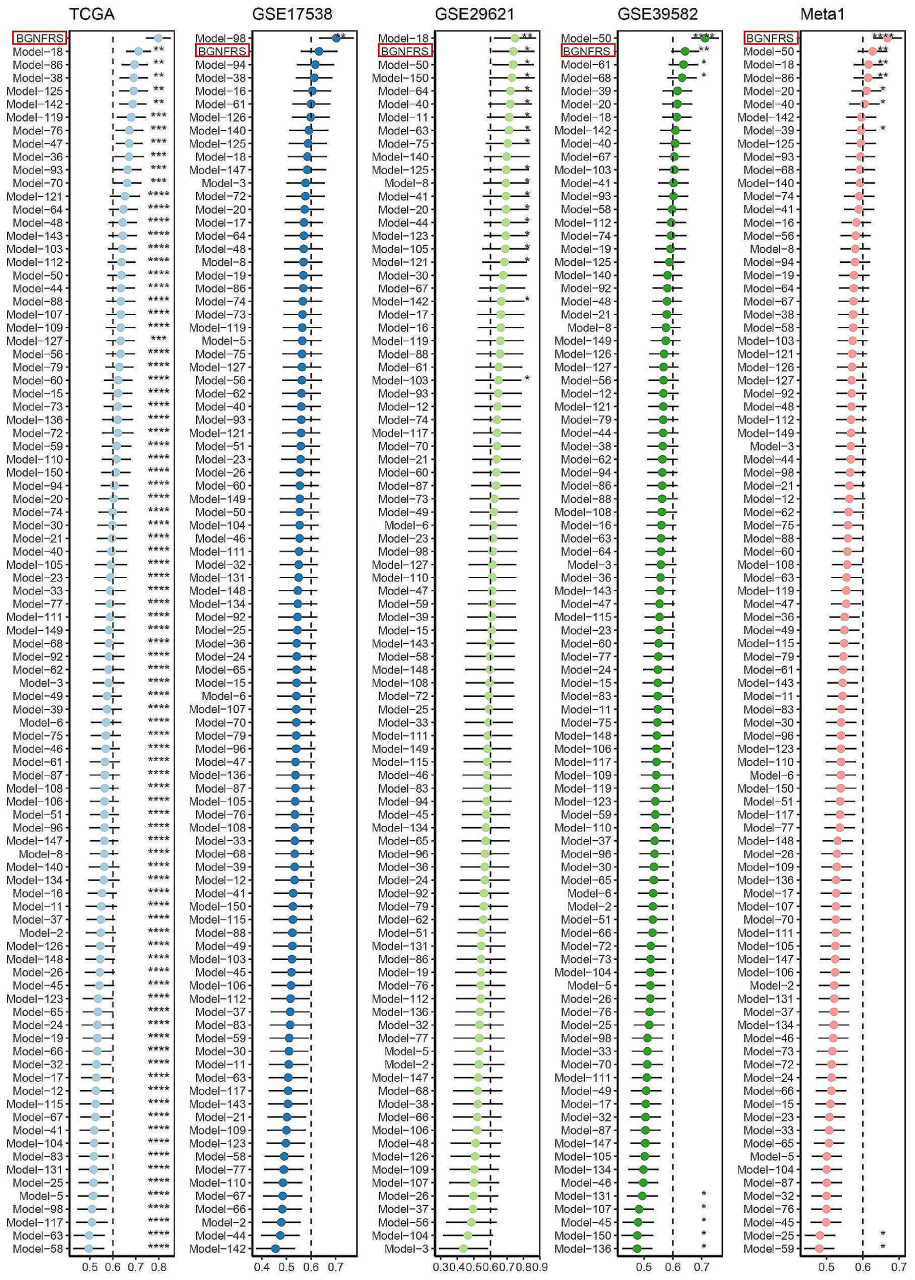
Comparison of the C-index between BGNFRS and the 92 published risk features

Numerous risk signatures for CRC have been published in the literature. In this study, a systematic search was conducted to identify 150 published risk signatures (Supplementary Table 4). Due to the lack of expression profiles for certain genes in the four datasets used in this study, only the gene expression profiles of 92 risk signatures were available. These 92 risk signatures were linked to diverse biological characteristics of CRC, including metabolism, immunity, autophagy, ferroptosis, and cell death. The C-index was calculated for these 92 risk signatures and compared to the BGNFRS. Notably, the BGNFRS ranked first in the TCGA cohort and second in the remaining three cohorts, but interestingly, it ranked first in the Meta1 cohort (Fig. 8). These findings underscore the robustness and high generalizability of the BGNFRS.

### BGNFRS is closely related to epithelial mesenchymal transition (EMT)

**Fig. 9:**
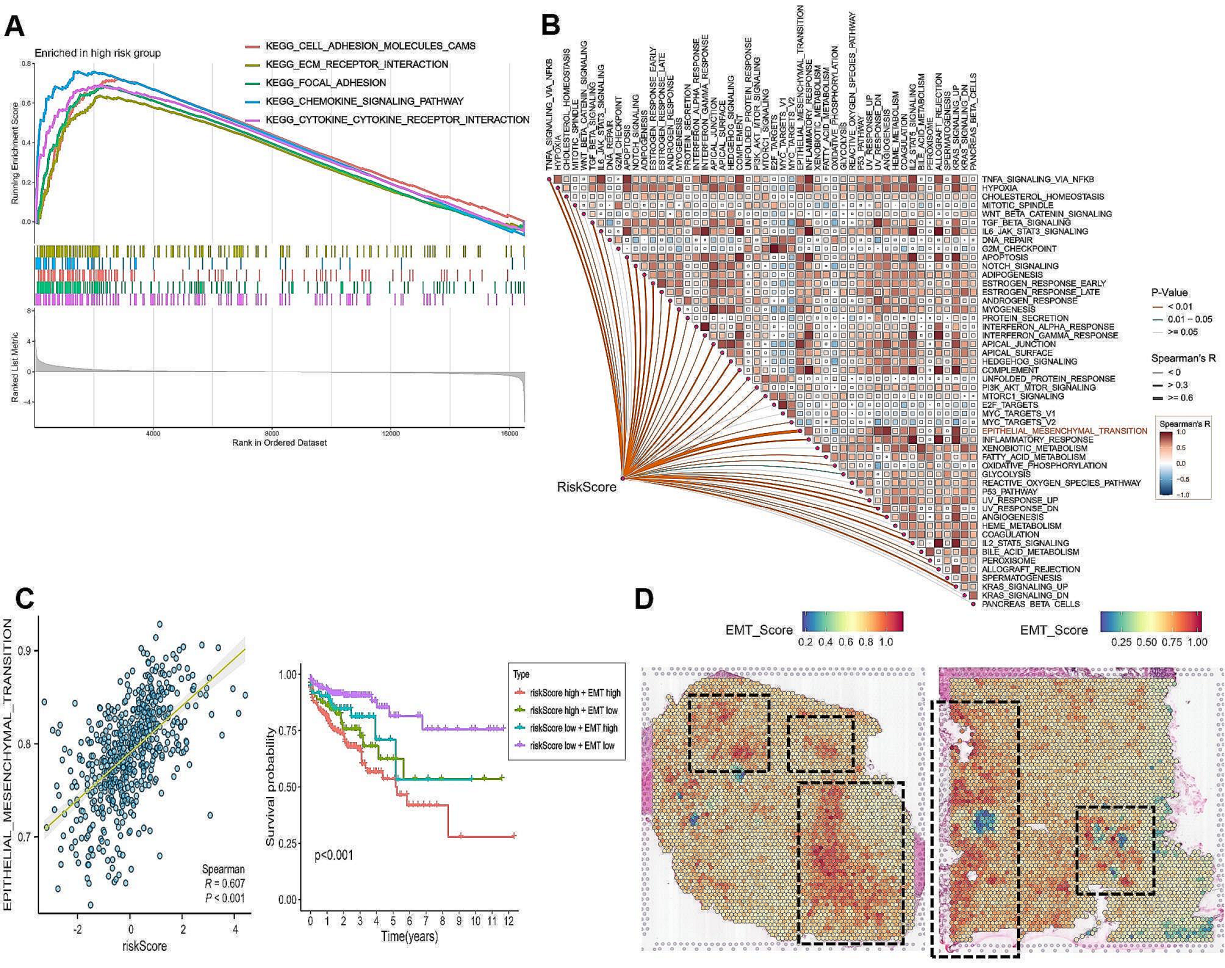
Association of BGNFRS with epithelial-mesenchymal transition (EMT) **A.** GSEA of the high-risk group of BGNFRS. **B.** Heatmap showing the correlation of BGNFRS risk score with hallmark pathways. **C.** Analysis of the correlation between BGNFRS risk score and EMT score. **D.** Spatial localization of EMT scores in two spatial tissue slices

To explore the biological characteristics of BGNFRS, this study conducted GSEA and GSVA based on the TCGA-CRC cohort. The analysis of GSEA using the KEGG gene set revealed that the high-risk group associated with BGNFRS exhibited significant enrichment in pathways related to cell adhesion and the extracellular matrix (Fig. 9A). The GSVA results, based on the HALLMARK gene set, demonstrated a positive correlation between BGNFRS and various hallmark pathways, with the strongest correlation observed in epithelial-mesenchymal transition (EMT) (*R* > 0.6, *P* < 0.05) (Fig. 9B-C). Further validation using spatial transcriptomics revealed that the spatial localization of EMT scores was largely overlapped with BGNFRS and BGN + Fib (Figs. 5G and 9D, and Supplementary Fig. 3D). Previous analyses have highlighted EMT as a key biological feature of BGN + Fib, with BGNFRS showing a positive association with EMT. Notably, EMT is recognized as a crucial factor contributing to metastasis in CRC patients [38], suggesting that higher BGNFRS risk scores are indicative of an increased likelihood of metastasis in CRC patients.

## Discussion

The recurrence and drug resistance of tumors have always been obstacles in the treatment of CRC [39]. Presently, the tumor microenvironment (TME) has shown promising potential in dynamically regulating cancer progression and influencing treatment outcomes [40]. CAFs, as the primary stromal component in the microenvironment [41], are potential targets for cancer therapy. However, most clinical trials targeting CAFs have yielded unexpected results, which may be attributed to their heterogeneity [6]. CAFs can undergo dynamic changes and even display contradictory biological functions as cancer progresses [6]. The specific roles of distinct CAF subtypes and their plasticity in interconversion remain largely unknown. Hence, we investigated CAF subpopulations in CRC and subsequently provided targeted prognosis assessment for precise CAF subtypes.

In this study, we observed that CAFs represent the primary cell population in direct communication with CRC cells and were systematically categorized. Subsequently, nine distinct CAF clusters (Fib_1–9) with different properties were identified. The study demonstrated that CAFs are not static but undergo dynamic changes during cancer development [42]. Currently, fibroblasts are commonly classified into three types, including MyFibroblasts CAF (myCAF), Immune regulatory/inflammatory CAF (iCAF), and Antigen presenting CAF (apCAF) [7]. Based on the surface markers and biological functional characteristics of these nine fibroblast subtypes, Fib_1/2/3/6/8 were classified as myCAF, while Fib_4/5/7/9 were classified as iCAF. Notably, Fib_3 exhibited the most substantial increase in the tumor group compared to the normal group and was predominant in the tumor group, with its prevalence escalating with CRC progression, particularly in TNM stage IV. Furthermore, time-series analysis revealed that Fib_3 is the terminal differentiation subtype of CAFs, suggesting its potential involvement in CRC advancement. To explore the key regulatory genes of Fib_3, a PPI network was constructed to obtain hub genes, and the BGN gene was ultimately identified as playing a crucial role in Fib_3. BGN, as a component of the extracellular matrix, primarily functions to maintain the structural integrity of the ECM [43]. Several studies have demonstrated a close association between the BGN gene and inflammation, with its overexpression observed in tumor tissues such as human pancreatic cancer and gastric cancer, where it plays crucial roles in tumor growth, adhesion, and invasion [44, 45]. A recent investigation highlighted the immunosuppressive nature of CAF-secreted BGN in triple-negative breast cancer [46]. Despite limited research on BGN in CRC currently, prior studies have shown a close correlation between BGN and CRC metastasis, EMT phenotype transition, and shorter survival time [47]. Notably, recent research has identified BGN derived from CAFs as a promising target for overcoming immunotherapy resistance [48]. In this study, considering the significant expression of BGN in Fib_3, and the similarity of BGN’s biological characteristics to Fib_3, Fib_3 is defined as BGN + Fibroblast (BGN + Fib).

Several subtypes of fibroblasts have been identified to play different roles in CRC. Qi et al. demonstrated that disrupting the interaction between FAP-positive fibroblasts and SPP1-positive macrophages can improve the efficacy of immunotherapy [4]. Zheng et al. showed that COL11A1 and INHBA-positive fibroblasts are adverse prognostic factors in CRC patients [49]. In the research conducted by Peng et al., MFAP5-positive fibroblasts were observed to influence the malignant microenvironment of CRC [50]. In our study, we identified BGN + Fib as an adverse prognostic factor for OS and RFS in CRC patients, which increased with the advancement of CRC stages (T, N, M, and stage). In vitro experiments revealed significantly higher expression of BGN in cancer-associated fibroblasts (CAFs) compared to normal fibroblasts (NFs). BGN + Fib was prevalent in CRC tissues but scarcely detected in normal colon tissues. Furthermore, downregulation of BGN expression in CAFs significantly reduced the migration and proliferation of CRC cells. Consequently, BGN + Fib acts as a driver in CRC.

Based on the previous analysis, we speculate the feasibility of constructing a CRC prognostic prediction model based on the relevant genes in BGN + Fib. CRC samples were stratified into high and low infiltration groups based on the median value of BGN + Fib infiltration. By utilizing WGCNA and machine learning, we ultimately constructed a BGN + Fib derived risk signature (BGNFRS) consisting of 17 risk-associated genes that exhibit stable prognostic prediction for CRC. Among these 17 genes, studies have shown that 15 genes are associated with CRC progression, including COMP, GPC1, POSTN, SLC2A3, CTHRC1, TNS1, INHBA, TIMP1, CAV1, AEBP1, CRYAB, THBS2, WNT5A, SPARCL1, and CALB2 [47, 51–63]. CPXM2 and CHPF have not been reported in CRC, but have been found to promote gastric cancer progression [64, 65]. The BGNFRS primarily originates from BGN + Fib and has been validated in spatial transcriptomic data. T, N, M, and stage are conventional tools for evaluating the prognosis and treatment of CRC patients [66]. Our BGNFRS was able to independently predict OS and RFS, surpassing the predictive capability of these factors. We also compared BGNFRS with 92 published CRC risk signatures and found that BGNFRS outperformed other risk signature based on the C-index. In order to improve the level of clinical application, we combined BGNFRS with different clinicopathological signature to build a nomogram prediction model. The nomogram prediction model showed good long-term prediction performance, and the prediction ability was significantly higher than other clinical prediction indicators.

BGNFRS may be involved in multiple signaling pathways that contribute to tumor initiation and progression, yet its potential role in CRC remains incompletely understood. Functional enrichment analysis revealed a high enrichment of the BGNFRS high-risk group in functional clusters related to epithelial-mesenchymal transition (EMT). EMT is a process in which cells lose their epithelial characteristics and acquire mesenchymal properties, ultimately increasing their motility and promoting an invasive phenotype [38]. EMT is believed to play a key role in the progression of various cancers, including CRC, by facilitating invasion and metastasis [67]. This observation indicates that BGNFRS-associated risk genes could potentially be involved in CRC metastasis and invasion, underscoring their promise as novel biomarkers warranting further exploration.

The BGNFRS model is readily reproducible through straightforward PCR amplification techniques, enhancing its practicality and aiding in clinical implementation. Nevertheless, notwithstanding the favorable predictive accuracy and potential clinical applicability of the BGNFRS model, it is essential to recognize its constraints. The retrieval keywords used in this study may not cover all signatures related to CRC risk, and have not included CRC risk-related signatures from other databases. Therefore, comparing BGNFRS with other published signatures is limited. Additionally, our data stem from retrospective analyses utilizing databases like GEO and TCGA, necessitating additional prospective investigations or the utilization of clinical samples and animal models to corroborate our discoveries. Secondly, certain individual samples within public datasets lack complete clinical information, potentially leading to bias in the data analysis outcomes.

## Conclusion

In conclusion, the study results suggest that BGN + Fib plays a significant role in driving CRC. This discovery enhances our comprehension of the involvement of CAF subpopulations in CRC and offers novel perspectives on devising therapeutic approaches targeting BGN + Fib. The BGN + Fib-derived BGNFRS exhibits promising predictive accuracy for both OS and RFS among CRC patients. This observation holds substantial importance for the management and prognostic evaluation of CRC.

### Electronic supplementary material

Below is the link to the electronic supplementary material.

Supplementary Material 1: Supplementary table1. Details of the dataset used in this study. Supplementary table2. Univariate Cox regression analysis of 167 genes. Supplementary table3. 17 BGNFRS genes and risk coefficient. Supplementary table4. 150 published risk signature.

Supplementary Material 2: Supplementary Figure 1. Comprehensive analysis of BGN. Supplementary Figure 2. The downregulation efficiency of BGN in CAFs. Supplementary Figure 3. WGCNA analysis, correlation analysis, and spatial transcriptomics validation of BGNFRS. Supplementary Figure 4. Differential analysis and multivariate Cox analysis of BGNFRS risk score with clinical and pathological features.

## Data Availability

The datasets presented in this study can be found in online repositories. These can be found in the GEO database (https://www.ncbi.nlm.nih.gov/geo), ArrayExpress (https://www.ebi.ac.uk/biostudies/arrayexpress) and The Cancer Genome Atlas (TCGA) (https://portal.gdc.cancer.gov). The original contributions presented in the study are included in the article/Supplementary Material. Further inquiries can be directed to the corresponding author.

## Abbreviations

BGN: biglycan
BGNFRS: BGN positive fibroblast derived risk signature
CRC: colorectal cancer
CAFs: cancer-associated fibroblasts
ECM: extracellular matrix
GEO: Gene Expression Omnibus
GSVA: Gene Set Variation Analysis
GSEA: Gene Set Enrichment Analysis
GO: Gene Ontology
KEGG: Kyoto Encyclopedia of Genes and Genomes
OS: overall survival
PPI: Protein Interaction Network
RFS: recurrence-free survival
TISCH2: Tumor Immune Single-cell Hub 2
TCGA: The Cancer Genome Atlas
WGCNA: Weighted Gene Coexpression Network Analysis
