# Supplementary material for: Identification of BGN positive fibroblasts as a driving factor for colorectal cancer and development of its related prognostic model combined with machine learning
Source: BMC Cancer. 2024 Apr 23;24:516. doi: 10.1186/s12885-024-12251-4 (PMC11041013; doi:10.1186/s12885-024-12251-4)
Supplement: Supplementary file 2 — Supplementary Material 2: Supplementary Figure 1. Comprehensive analysis of BGN. Supplementary Figure 2. The downregulation efficiency of BGN in CAFs. Supplementary Figure 3. WGCNA analysis, correlation analysis, and spatial transcriptomics validation of BGNFRS. Supplementary Figure 4. Differential analysis and multivariate Cox analysis of BGNFRS risk score with clinical and pathological features. [file 12885_2024_12251_MOESM2_ESM.docx]

**Supplemental figure**

**
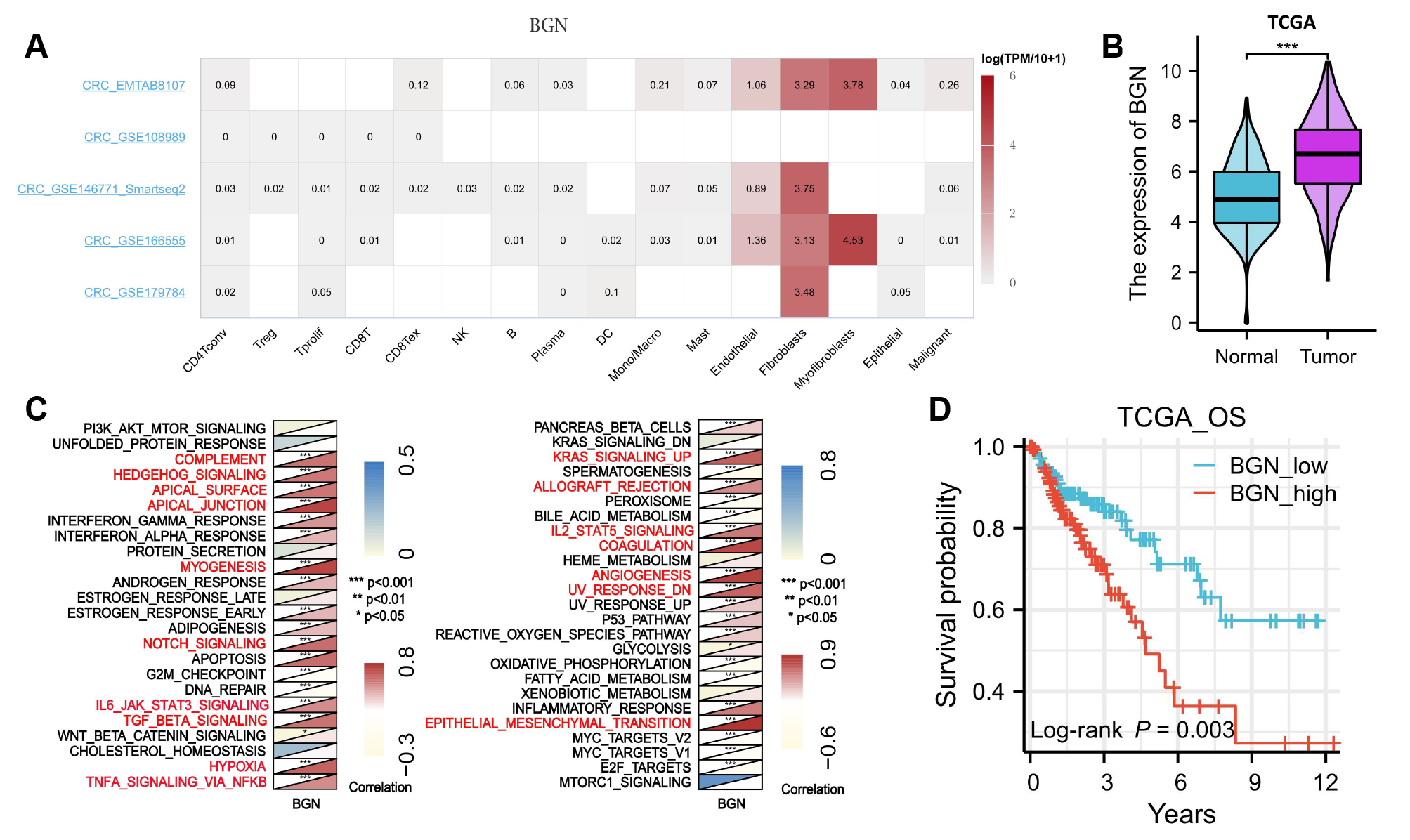
**

**Supplementary Figure 1.** Comprehensive analysis of BGN.

1. Expression heatmap of BGN in five single-cell datasets. B. Differential expression analysis of BGN in the TCGA-CRC cohort. C. Correlation analysis between BGN and pathway scores based on GSVA (Red font indicates correlation greater than 0.6). D. Survival analysis between high and low expression groups of BGN.


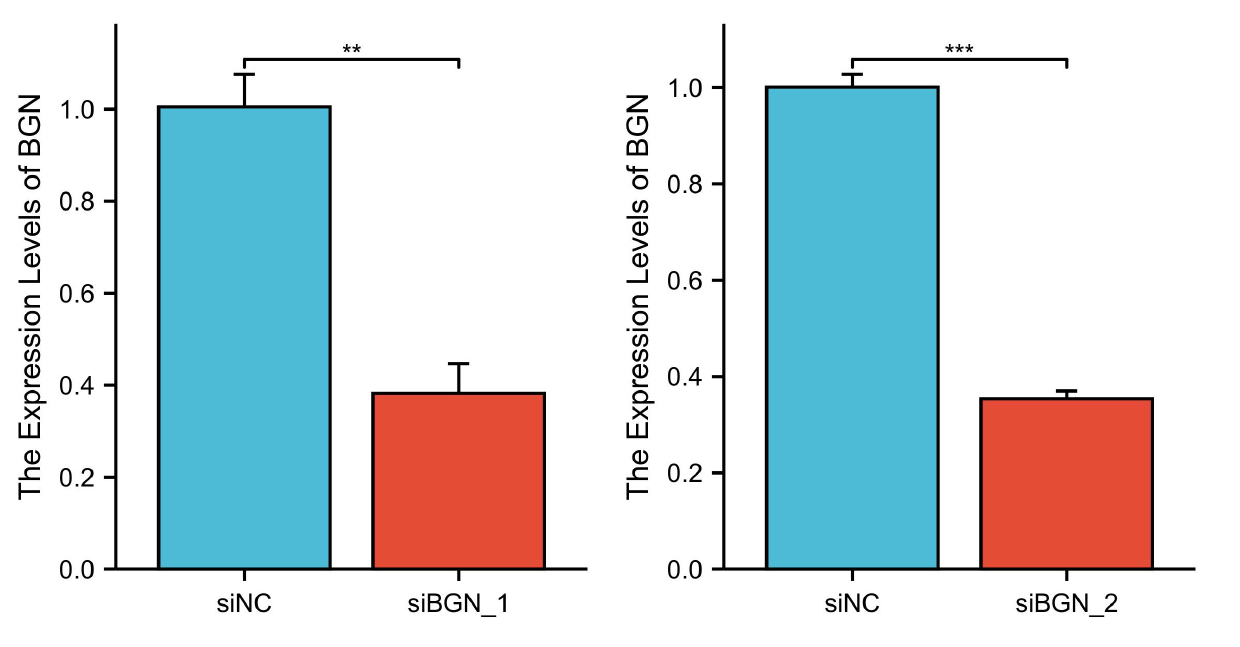


**Supplementary Figure 2.** QRT-PCR results showed the downregulation efficiency of BGN in CAFs..


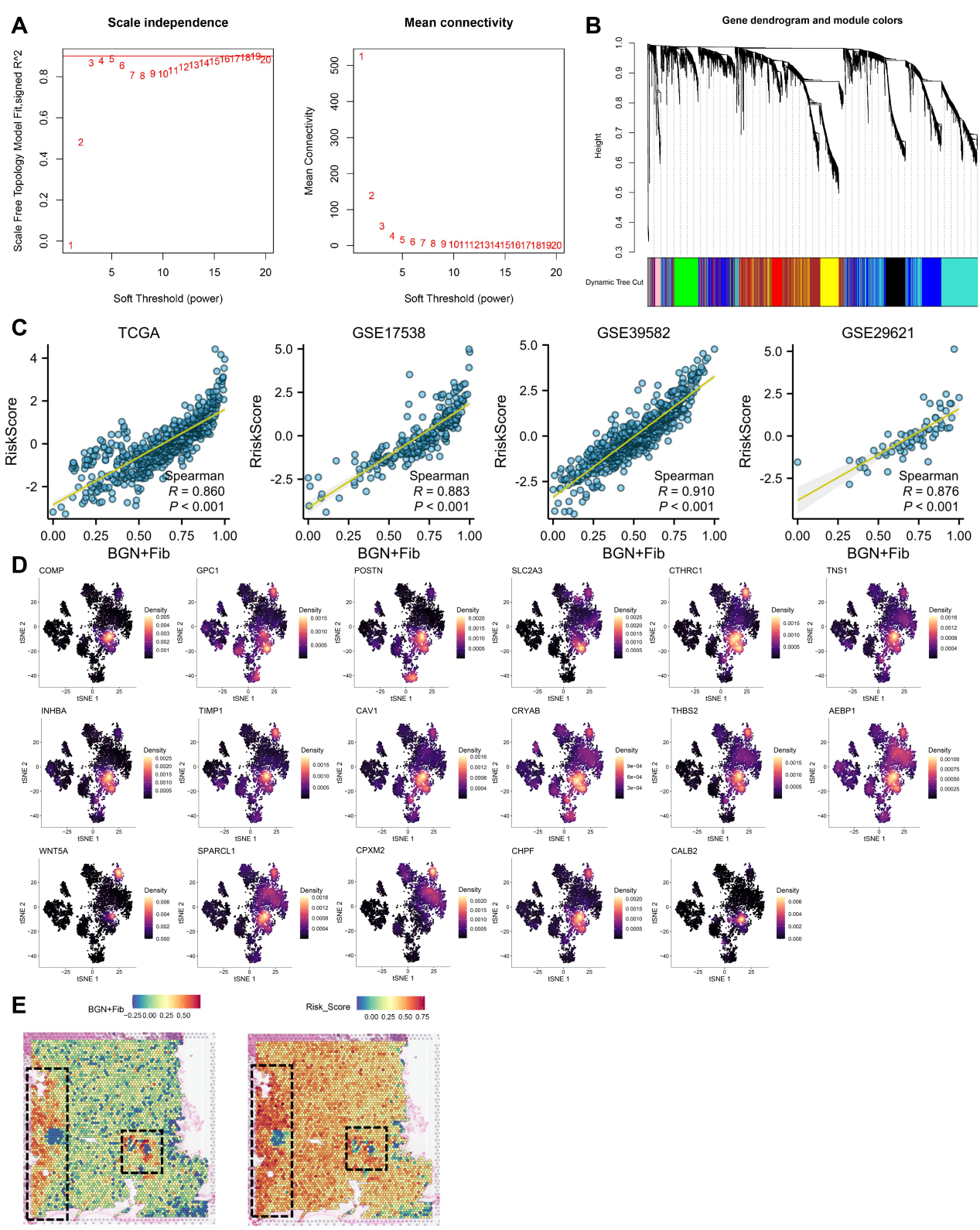


**Supplementary Figure 3.** WGCNA analysis, correlation analysis, and spatial transcriptomics validation of BGN+Fib-related risk signature (BGNFRS).

1. B. Calculation of WGCNA soft threshold and generation of corresponding dendrogram for high BGN+Fib infiltration group and low BGN+Fib infiltration group. C. Correlation analysis between BGNFRS risk score and BGN+Fib. D. The tSNE plot shows the expression of 17 signature genes in the fibroblast subpopulation. E. Spatial localization of BGN+Fib and BGNFRS in spatial transcriptomics.


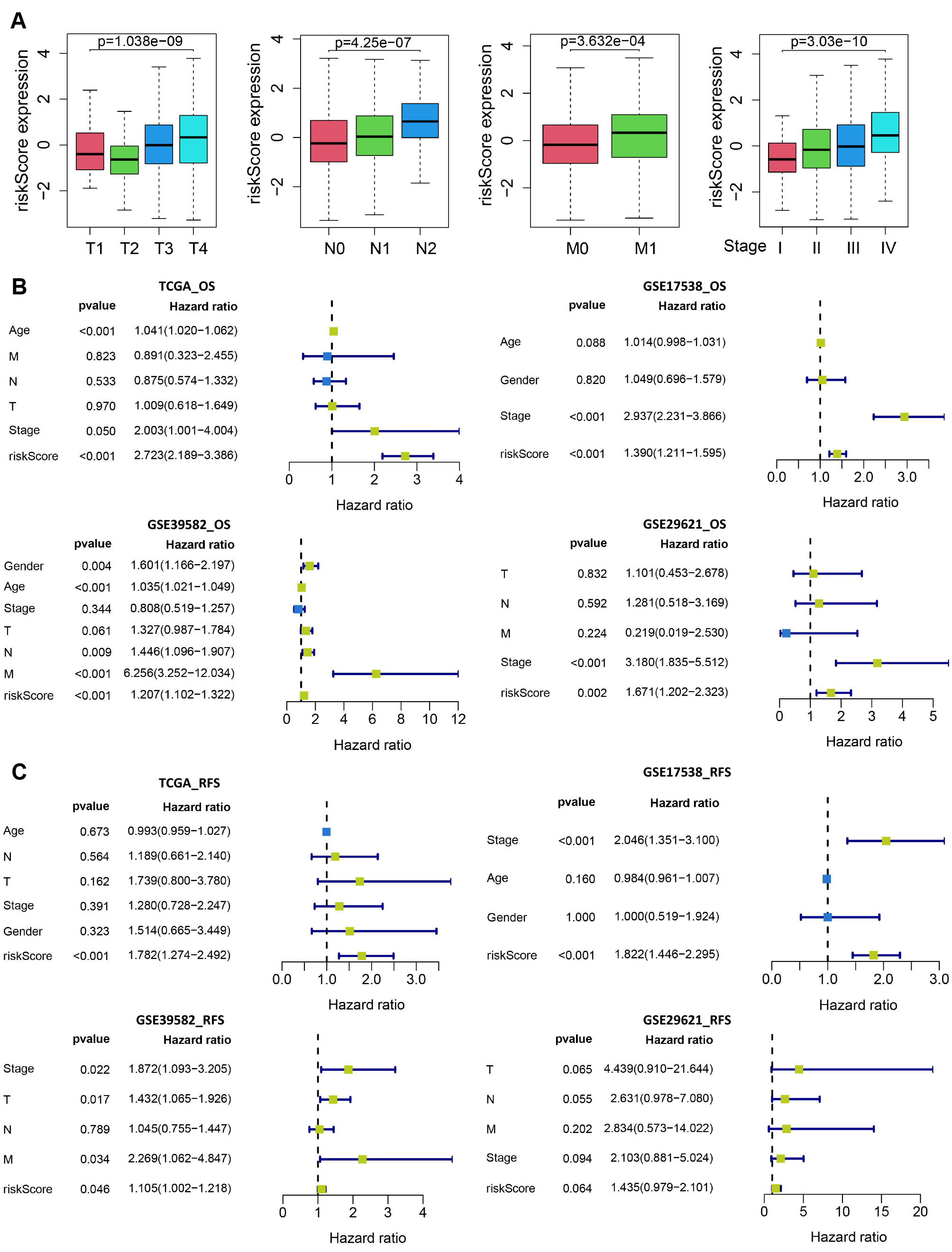


**Supplementary Figure 4.** Differential analysis and multivariate Cox analysis of BGNFRS risk score with clinical and pathological features.

1. Differential analysis of BGNFRS risk score with clinical and pathological features. B-C. Multivariate Cox analysis combining OS/RFS with CRC clinical and pathological features.
